# Signal-On Fluorescent Imprinted Nanoparticles for Sensing of Phenols in Aqueous Olive Leaves Extracts

**DOI:** 10.3390/nano10061011

**Published:** 2020-05-26

**Authors:** Ada Stavro Santarosa, Federico Berti, Martina Tommasini, Antonella Calabretti, Cristina Forzato

**Affiliations:** Dipartimento di Scienze Chimiche e Farmaceutiche, Università degli Studi di Trieste, via Giorgieri 1, 34127 Trieste, Italy; adastavro@gmail.com (A.S.S.); fberti@units.it (F.B.); martina.tommasini28@gmail.com (M.T.); ANTONELLA.CALABRETTI@deams.units.it (A.C.)

**Keywords:** imprinted nanogel, phenols, fluorescence, olive leaf extracts

## Abstract

The activation of signals in fluorescent nanosensors upon interaction with their targets is highly desirable. To this aim, several molecularly imprinted nanogels have been synthetized for the recognition of tyrosol, hydroxytyrosol and oleuropein in aqueous extracts using the non-covalent approach. Two of them contain fluorescein derivatives as co-monomers, and their fluorescence emission is switched on upon binding of the target phenols. The selection of functional monomers was previously done by analyzing the interactions by nuclear magnetic resonance (NMR) in deuterated dimethylsulfoxide (DMSO-*d*_6_) of the monomers with tyrosol and hydroxytyrosol. Polymers were synthetized under high dilution conditions to obtain micro- and nano-particles, as verified by transmission electron microscopy (TEM). 1,4-Divinylbenzene (DVB) was used in the fluorescent polymers in order to enhance the interactions with the aromatic ring of the templates tyrosol and hydroxytyrosol by π-π stacking. The results were fully satisfactory as to rebinding: DVB-crosslinked molecularly imprinted polymers (MIPs) gave over 50 nmol/mg rebinding. The sensitivity of the fluorescent MIPs was excellent, with LODs in the pM range. The sensing polymers were tested on real olive leaves extracts, with very good performance and negligible matrix effects.

## 1. Introduction

The development of simple sensors able to detect and quantify specific analytes is a continually expanding sector. Among the several recognition elements that are necessary to build up a sensor, the molecularly imprinted polymers (MIPs) approach has received great attention in recent years toward obtaining sensing nanomaterials, since it is specific, cheap and stable to different conditions, albeit with lower sensitivity and selectivity in comparison to antibodies or other recognition elements [1]. Due to their enzyme and antibody mimicking approach, the “lock and key” mechanism, MIPs are usually defined as biosensors. Two different approaches can be used in preparing MIPs, the covalent and the non-covalent one. In the covalent approach, the imprinted molecule is covalently attached to the polymerizable molecule by using reversible covalent binding, and a chemical cleavage is necessary at the end of polymerization to remove the template. The most frequent approach is the non-covalent imprinting due to its simplicity and accessibility. In this approach, a specific binding site is built by self-assembly of the functional monomer, the chosen template and a cross-linker. The removal of the template in this case is by solvent extraction. MIPs can be used in several fields such as in extraction processes, as they are used as stationary phases for chromatography and SPE (solid phase extraction), in detection of small molecule analytes, in surface covering of gold and silver nanoparticles to use in Raman SERS (surface enhanced Raman scattering) or in drug delivery [2]. Regarding detection, these particular biosensors can be classified into electrochemical, optical or other sensors, depending on the transducer, and one of the main application fields is food safety. In fact, MIPs have been prepared for the detection of natural toxins, residues of pesticides, veterinary drugs, environmental contaminants from soil and water, abused additives and contaminants from processing and packaging [3]. 

**Figure 1:**
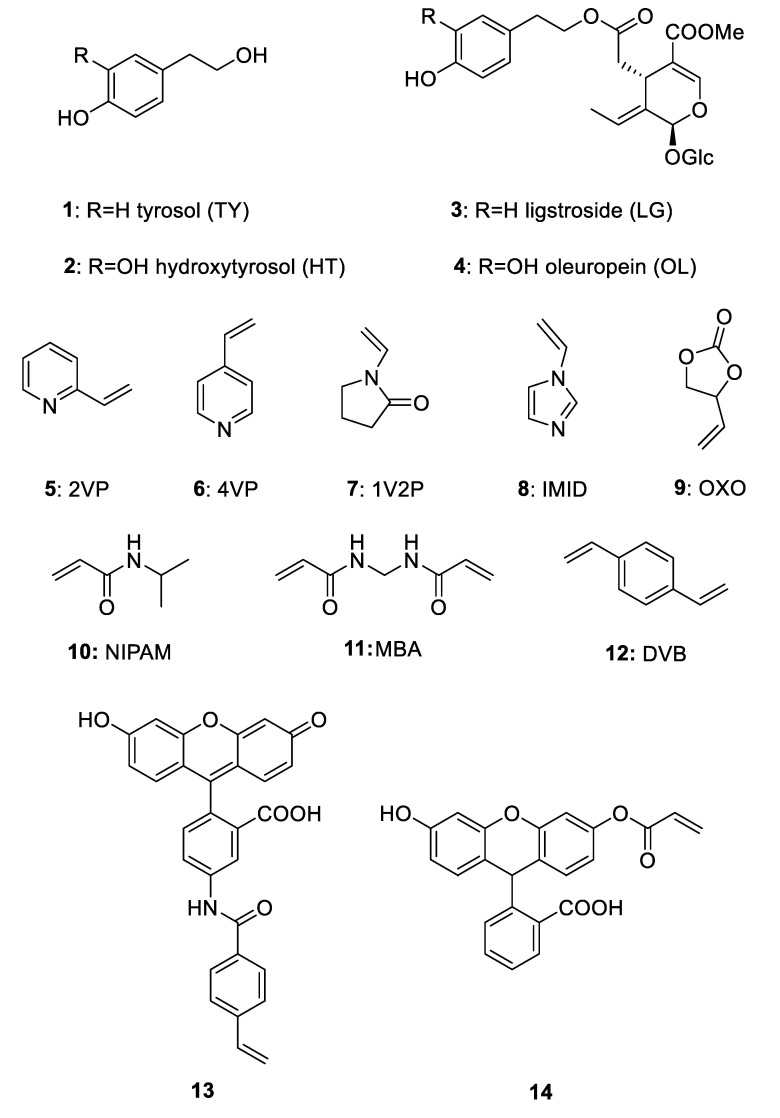
Chemical structures of tyrosol (TY) **1**, hydroxytyrosol (HT) **2**, ligstroside (LG) **3**, oleuropein (OL, Glc: glucose) **4** and of monomers **5**–**9**, co-monomers **10, 13, 14** and cross-linkers **11, 12**.

To date, MIPs for sensing food analytes have often a relatively low sensitivity and suffer from matrix effect, but the fluorescence sensing technology has demonstrated to have the advantages of specific recognition and specific adsorption of molecular imprinting, together with a high sensitivity and a high selectivity. Fluorescent materials with both these characteristics are therefore important in the rapid detection of food quality [4]. Moreover, the design of an imprinted nanomaterial capable to activate a signal such as fluorescence emission, rather than quenching, would be very helpful to improve sensitivity. Fluorescent imprinted polymers usually have been obtained with naphtalimide fluorophores, quantum dots, and other sensing molecules as fluorescein. Very few MIPs containing fluorescein give an enhancement of emission of the fluorescent signal upon interaction with the target [5,6,7,8]. To this end, in the present work, the non-covalent approach has been exploited to prepare several imprinted nanomaterials (nanogels), able to capture specifically tyrosol (TY) **1** and hydroxytyrol (HT) **2** (Figure 1). 

The target molecules are important analytes in the quality evaluation of olive oil and of olive leaves extracts. They represent key compounds in the beneficial effect of the Mediterranean diet, which has a strong influence in preventing cardiovascular diseases, and consumption of olive oil is one of the factors contributing to the healthiness of this diet. Clinical studies proved that substituting saturated fatty acids with the monounsaturated ones present in olive oil, which is particularly rich with oleic acid, a decrease in LDL (low density lipoprotein) plasmatic concentration is observed. Recently, also the minor components of olive oil were studied in protecting the oxidative stress of the LDL, attributing to the phenols and polyphenols the antioxidant properties [9]. These compounds are present not only in the olive oil but also in olive leaves extracts, both aqueous and alcoholic, which have been used in traditional medicine from centuries to treat gastro-intestinal inflammations, urinary and respiratory inflammations or as diuretics or antihypertensive drugs [10]. TY **1**, HT **2** and their corresponding derivatives ligstroside **3** and oleuropein (OL) **4** are the most abundant polyphenols present in olive oil and aqueous olive leaves extracts as secondary metabolites present in the fruits and leaves of the olive tree (Figure 1). These compounds are produced by the plant for defense against pathogen organisms and insects and their concentration in the fruit and the leaf depends on several factors, such as type of cultivar, geographical position, climate changes and other external factors that could affect the biosynthesis [11]. The beneficial effects of olive polyphenols have been recently recognized by the EFSA (European Food Safety Authority), which in 2011 verified the cause and effect relationship between the assumption of polyphenols with diet and the prevention of lipoperoxidation of LDL. In particular, a daily intake of at least 5 mg of polyphenols (tyrosol, hydroxytyrosol and their derivatives) will provide these beneficial effects (Commission Regulation EU 432/2012). The development of a simple method for the identification and quantification of these polyphenols can be of great interest for the olive oil producers and for the industries involved in the production of nutraceutical products based on olive extracts. Herbal teas are traditionally used among Mediterranean people to cure certain diseases and the potential health benefits recently reported [12,13,14,15], such as antioxidant, hypoglycemic, antihypertensive, antimicrobial and antiatherosclerotic effects of olive leaves, stimulated the research in the analytical sector and the development of new technologies for the identification of these compounds [16].

The identification and quantification of the polyphenols present in olive oil are usually performed by high performance liquid chromatography (HPLC), when standards are available, or by HPLC coupled with mass spectrometry (MS) [17]. The analytic procedure, although simple, is rather time consuming and expensive since highly costly chromatographic techniques are required.

The necessity for the development of a rapid system in the identification of the most important phenols present both in olive oil and in aqueous extracts of olive leaves is becoming urgent in the agro-economical sector. The present work focuses on the development of sensing elements based on the molecular imprinting method to identify and quantify the polyphenols of interest. With this purpose in mind, different monomers, co-monomers and cross-linkers have been tested in order to achieve polymers with the optimal properties to establish the right equilibrium between the release and capture of the templates. As a first attempt, commercial monomers **5**–**9** were used in the MIPs synthesis, choosing *N*-isopropylacrylamide (NIPAM) as the co-monomer and *N,N′*-methylenebisacrylamide (MBA) as the cross-linker as they showed good results in our previous works [18,19] (Figure 1). Taking advantage of the higher sensitivity and selectivity in general of fluorescent MIPs, the fluorescent co-monomer **13** and **14**, obtained by a literature synthetic procedure, were used. To improve the properties of the fluorescent MIPs, DVB was chosen as the cross-linker as explained in Section 3.4. Although the synthetized MIPs were built using TY and HT as the templates, they have been tested also for capturing OL **4**, as this is the major phenol present in olive leaves extracts. These fluorescent MIP nanogels have been designed as signal-on sensors for the direct detection of phenols in olive leaves extracts by fluorimetry. The fluorescein based co-monomers used **13** and **14** were used with the aim of obtaining activation of their emission upon binding as already observed in the literature for other templates.

## 2. Materials and Methods 

### 2.1. Chemicals

Tyrosol (TY) **1**, hydroxytyrosol (HT) **2** and oleuropein (OL) **4** were purchased from Carbosynth (Compton, UK). 2-Vinylpyridine (2VP) **5**, 4-vinylpyridine (4VP) **6**, 1-vinyl-2-pyrrolidinone (1V2P) **7**, 1-vinylimidazole (IMID) **8**, 4-vinyl-1,3-dioxolan-2-one (OXO) **9**, *N*-isopropylacrylamide (NIPAM) **10**, *N,N′*-methylenebisacrylamide (MBA) **11**, 1,4-divinylbenzene (DVB) **12**, fluoresceine-O-acrylate and 2,2′-azobisisobutyronitrile (AIBN) were from Sigma-Aldrich (Milano, Italy). Fluorescent monomer **13** was synthetized according to the literature [8]. AIBN was recrystallized according to the literature [18].

### 2.2. Instrumentation

NMR spectra (500 MHz) were recorded on a Varian 500 spectrometer (Palo Alto, CA, USA). HPLC analyses were run on an Agilent series 1100 liquid chromatograph (Santa Clara, CA, USA). Fluorimetric titrations were performed on a Perkin Elmer Luminescence spectrometer (Waltham, MA, USA) at 25 °C with a square quartz cuvette of 5 mm optical path. TEM images were recorded with a Camera Olympus QUEMESA (Tokyo, Japan) and software RADIUS (EMSIS) (Münster, Germany) on a TEM images Philips EM208 (Amsterdam, The Netherlands) at 100 KV using a 200 mesh copper grid with carbon film.

### 2.3. ^1^H-NMR Titrations

For each titration, a 6.7 mM solution of the template (**1** and **2**) and a 1.0 M solution of each monomer **5**–**9** were prepared. In a NMR tube, 2.5 μL aliquots of the monomer solution (2.5 μmol, 0.5 equiv.) were added to 750 μL of the template solution (5.0 μmol) progressively, until a total amount of 10 equiv. were added. The ^1^H-NMR spectra of the resulting solutions were recorded after every addition. The same titrations were performed with templates **1**,**2** and fluorophore monomers **13**,**14**.

### 2.4. Synthesis of Molecularly Imprinted Polymers (General Procedure)

The functional monomer (1 equiv.) and the template (1.2 equiv.) were stirred in anhydrous DMSO for 1 h at room temperature in a vial. Subsequently, a solution of *N*-isopropylacrylamide (1 equiv.), cross-linker (4.6 equiv.) and 2,2′-azobisisobutyronitrile (2 equiv.) in DMSO was added and the vial was flushed with argon and sealed with a crimp cap. 60 equiv. of DMSO were used in total, divided between the two solutions. The polymerizing mixture was kept at 70 °C for 24 h.

Each polymer was synthetized both in presence of the template molecule, leading to MIP particles, and without the template, leading to NIP (non-imprinted polymers). The resulting clear solutions were dialyzed (cut off 3.5 kDa) against water for 2 days, against a MeOH:AcOH (8:2) mixture for 2 days and against water for 5 additional days, changing the solvent twice a day.

Finally, the solutions were freeze-dried giving a fluffy solid. The composition of the polymerization mixtures is reported in Table 1.

### 2.5. Transmission Electron Microscopy

Lyophilized MIPs were suspended in distilled water (0.2 mg mL^−1^) and sonicated for 30 min. Each solution was then diluted 40 times with distilled water and sonicated for 1 h. A drop of this solution was then placed on an amorphous carbon coated grid, and left to dry at room temperature for one night; TEM images of the MIPs were finally recorded. 

### 2.6. Rebinding Tests

A mixture of polymer (2 mg) (MIP or NIP) and the corresponding template (100 μM) in 2 mL of water was kept under stirring at room temperature. 400 μL aliquots were taken at different times between 10 min and 24 h and centrifuged (15,100 rpm for 10 min). The supernatant was analyzed by HPLC to quantify the residual free template concentration. A Kinetex C18 250 × 4.6 mm 5 μm 100 Å (Phenomenex) column was used with a column guard and a 20 μL loop and a UV detector set at 220 nm. The flow was set to 1 mL/min. The eluent was solvent A: H_2_O + 0.05% TFA and solvent B: CH_3_CN + 0.05% TFA in 85:15 ratio for analysis of TY and HT, while a gradient was used for the analysis of OL (10 min 85% A and 15% B, 10 min 40% A and 60% B, 10 min 85% A and 15% B).

Quantitative determination was performed by HPLC using the calibration curves of TY, HT and OL reported in Supplementary Materials (Figures S1 and S2). Standard stock solutions were prepared in water at 10, 30, 50, 80 and 100 μM concentrations. Each solution was analyzed by HPLC in triplicate and the mean value of each solution was used for the calibration curve.

The concentration of the free template at each time was calculated as follows:

(1) $${\left[\mathrm{Templ}\right]}_{t}=\frac{{\mathrm{Area}}_{t}}{{\mathrm{Area}}_{t0}}\times {\left[\mathrm{Templ}\right]}_{t0},$$

where ${\left[\mathrm{Templ}\right]}_{t}$ is the concentration of the template at time *t*, ${\mathrm{Area}}_{t}$ is the area (mV·s) of the chromatogram peak of the template at time *t*, ${\mathrm{Area}}_{t0}$ is the area (mV·s) of the chromatogram peak of the template at the beginning, and ${\left[\mathrm{Templ}\right]}_{t0}$ is the concentration of the template at the beginning (100 μM). The concentration of the template bound to the polymer (nmol/mg) were calculated as follows:

(2) $$\left[\mathrm{Templ}\right]=({\left[\mathrm{Templ}\right]}_{t0}-{\left[\mathrm{Templ}\right]}_{t})\times \frac{V}{M},$$

where [Templ] is the concentration of the template bound to the polymer, ${\left[\mathrm{Templ}\right]}_{t}$ is the concentration of the template at time *t*, ${\left[\mathrm{Templ}\right]}_{t0}$ is the concentration of the template at the beginning (100 μM), *V* is the volume (mL) of the solution at the beginning and *M* is the mass (mg) of polymer used in the rebinding test.

The imprinting factor (IF) was calculated as the ratio [Templ]_MIP_/[Templ]_NIP_.

Cross reactivity tests with TY and HT were performed in the same manner at 50 and 100 μM ligand.

### 2.7. Fluorimetry

A 1 mg/mL mother solution in mQ water of the polymers was prepared and diluted 1:400 to obtain a final concentration of 2.5 μg/mL. For each titration, an initial volume of 800 μL of the 2.5 μg/mL colloidal solution was placed in a fluorimetry quartz square cell with 1 cm optical path, and 8 μL of each solution of the corresponding template were subsequently added. The measures were carried out after 10 min incubation at 20.0 °C, collecting the emission spectra between 500 and 600 nm, at 480 nm excitation. Titration curves were obtained by triplicate measures at 515 nm emission wavelength, corresponding to the maximum in the emission spectra, with excitation and emission slits set at 3 and 7 nm respectively. 

### 2.8. Olive Leaves Extract

200 g of fresh olive leaves were frozen in liquid nitrogen, ground into a mortar and then stirred in 2 L of distilled water at a temperature between 70 and 90 °C for 4 h. The mixture was filtered with a paper filter under vacuum and the volume was reduced to 1 L. The obtained solution was filtered with a PTFE 0.45 μm filter and successively with a PTFE 0.2 μm before injection on HPLC. HPLC analysis was carried out as described in rebinding test section. 

## 3. Results and Discussion

### 3.1. Design of the Imprinted Materials

The interactions of the template with the functional monomers is the key step in formulating the MIP composition, based on weak non-covalent interactions such as hydrogen bonding, ionic interactions or van der Waals forces depending on the structure of the template. Both TY and HT have an aromatic ring that could be involved in π-π stacking with other aromatic functions or unsaturated systems, while the phenolic groups could act as donors or receptors for hydrogen bonds. In order to design imprinted polymers capable of both satisfactory rebinding and sensitive detection, we have chosen to explore the effect of the composition at three levels. 

First, we have selected non-fluorescent functional monomers capable of establishing significant interactions with the targets. To this end, compounds **5**–**9** reported in Figure 1 were chosen. Such monomers are widely used in the preparation of imprinted polymers and are in principle capable to establish the required interactions by hydrogen bonding. We have also chosen NIPAM **10** as a co-monomer. It is demonstrated that NIPAM usually does not interact with the template in polar solvents as DMSO and water, but acts as a stabilizer of the polymeric structure [20].

Second, we have considered two opposite approaches in the selection of the cross-linker. On one side we have chosen MBA **11**, a very common and minimal length cross-linker, capable in principle to contribute to ligand binding by hydrogen bonding. On the other side we have also considered a cross-linker fully capable of further hydrophobic interactions with the aromatic systems of the phenols, namely DVB **12**.

Third, as to the fluorescent co-monomers, we have focused on fluorescein. The literature reports several examples of MIPs utilizing this fluorophore as signaling label. In most cases, quenching of its emission has been observed upon rebinding of the template [21,22]. However, Wren and colleagues have described in 2014 a MIP for the recognition of cocaine with activation of fluorescein emission upon binding, using as the monomer a 4-vinylbenzamide derivative of fluorescein **12** [8]. The authors explain the increase of fluorescence on the basis of a proton transfer from the carboxylic group of fluorescein to the amino group of cocaine, that enhances the fraction of the more fluorescent fluorescein anion. 

**Figure 2:**
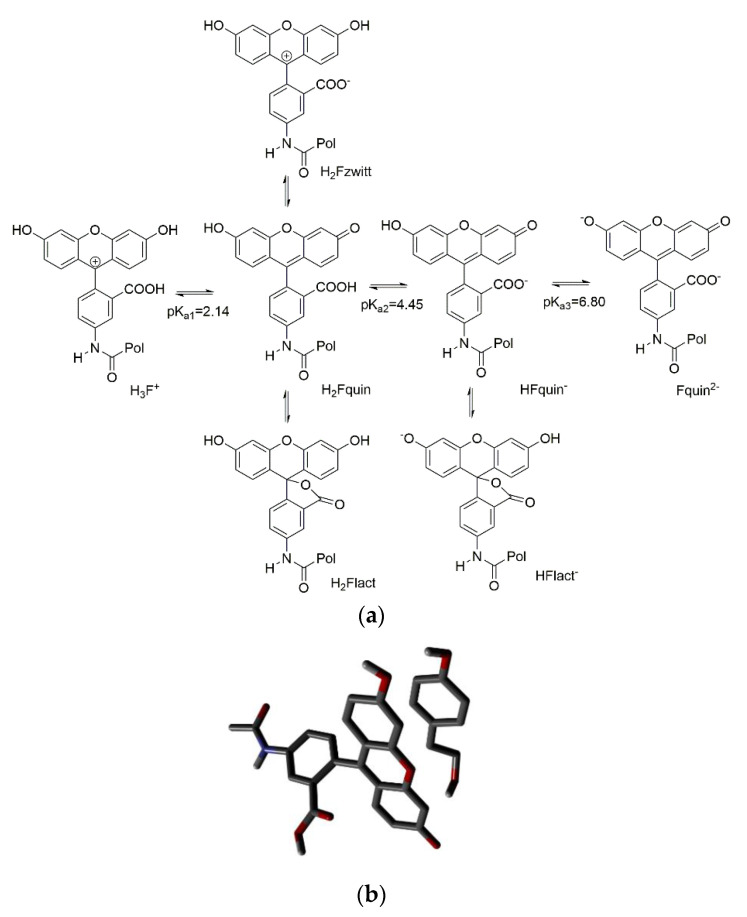
(**a**) Equilibria of fluorescein in water solution [23]; (**b**) optimized geometry of the complex of tyrosol and the H_2_Fquin form of fluorescein.

However, in water fluorescein undergoes to a rather complex equilibrium between tautomers and protonated/deprotonated forms (Figure 2a) [23].

We reasoned that such equilibria could be shifted by the presence of the template even if it was not capable of proton transfer. The nonfluorescent lactonic tautomers H_2_Flact and HFlact^-^ are in equilibrium with the fluorescent ones H_2_Fquin and HFquin^-^ which are present in solution up to neutral pH. An aromatic ligand as our phenols could favorably shift the equilibrium towards the fluorescent tautomers. To evaluate this possibility, we have carried out DFT calculations at the B3lyp-Df-6.31G** + level in a pcm water model. The lactone tautomer H_2_Flact results more stable than the open acid H_2_Fquin by 10.44 KJ/mol, in agreement with the experimental observations [23]. A model of the π-stacked complexes of tyrosol and the two tautomers was in favor of the open acid complex (Figure 2b) by 13.2 KJ/mol thus giving an indication of a possible shift towards the fluorescent form in the presence of tyrosol. A similar behavior was obtained also by modelling the complexes of tyrosol with the monoanionic forms of fluorescein. We have therefore considered two fluorophore monomers, namely **12** as proposed by Wren [8], and *O*-acryloyl-fluorescein **13**.

The designed imprinted polymers are aimed at sensing phenols in aqueous extracts of olive leaves, where the most important derivative is oleuropein. However, this compound is scarcely available, and moreover it is likely unstable under radical polymerization conditions, due to the presence of the elenolic acid skeleton which contains an activated double bond. For this reason, we have chosen to carry out polymerizations utilizing as templates the cheap and available TY and HT, which are part of the OL and ligstroside structures. The synthesized polymers will be tested also for the detection of the more complex OL.

A very common protocol in the design and synthesis of MIPs involve a preliminary evaluation of the interactions between the template and potential functional monomers, carried out by NMR titration, and we have also followed this way in the past. However, there is a number of reasonable questions about the usefulness of this approach, as the interactions between the free molecules maybe very weak in the absence of proximity effects operating inside the MIP structure. Moreover, NMR titrations are carried out at very high concentration of the partners and at room temperature, while polymerization is obtained at 70 °C and under high dilution conditions. We have therefore decided to exploit the present work also to evaluate such point in our case, and we have both carried out NMR titrations and performed MIP synthesis on the set of functional monomers **5**–**9**. The interaction between the two templates and each functional monomer was studied by ^1^H-NMR in DMSO-*d*_6_ by progressive addition of a solution of the functional monomer to a solution of the template in order to evaluate the variation of the chemical shift of the single protons of the templates. In the supplementary material are reported selected changes of NMR peaks upon titrations for the most relevant cases, and overall changes after the addition of 10 equivalent functional monomers (Figures S3–S12). For TY, the most relevant variations in the chemical shift were observed with 4VP on all the protons of template. Additionally, the corresponding isomer 2VP determined a variation of all protons but to a minor extent. IMID determined a variation for the only OH protons, while 1V2P and OXO did not show any significant interaction. Similarly, the major variations in the chemical shifts of all protons of HT were observed during titrations with 4VP, although significant interactions were evidenced also with the 2VP isomer. As already observed for TY, monomers IMID and OXO did not interact with HT while IMID determined a variation for the only OH protons. Interactions with the fluorescent co-monomers **13** and **14** showed significant variations for protons C2′-OH and C4-OH for both monomers in the case of TY although higher intensity was detected with co-monomer. The same variations were observed for HT with the addition of variation of C3-OH. 

### 3.2. Synthesis and Characterization of the Polymers

The polymers were synthetized according to a procedure already described by us, in order to obtain micro- and nano-particles under high dilution conditions [18]. To prepare non-fluorescent polymers for the first evaluation round, monomers **5**–**9** were used in concentration of 1% and DMSO was chosen as the porogen solvent. NIPAM was used as the co-monomer and MBA as the cross-linker while AIBN was chosen as the radical initiator. The co-monomer was present in 15 mol % to enhance the yield of the polymerization and to enhance the stability of the polymer. 

Yields of the MIPs were in most cases satisfactory, up to 95% depending on the monomer used (Table 2). The higher yield resulted for the materials obtained from 4VP. When 4VP is used, together with co-monomers **13** and **14** and DVB as the cross-linker, as in the fluorescent MIPs, the yields are lower, probably due to the presence of the less reactive DVB. Among the other monomers used, 1V2P and IMID lead to the higher yields while the lower yield was for the OXO polymers. These results do not reflect the NMR observation but seem to be related to the intrinsic reactivity of the monomers. In fact, OXO is the less chemically reactive one. The corresponding non-imprinted polymers (NIPs) were also prepared in the same way, but in the absence of templates. The yields obtained for NIPs are unusually high in comparison with those of the corresponding MIPs. It is known in the literature that NIPs synthesis is very often less satisfactory as we have also verified in our experience. Nevertheless, in this case the high yield of NIPs could be explained by the absence of the template since the templates used, due to their structure, could inhibit to some extent the radical polymerization. 

**Figure 3:**
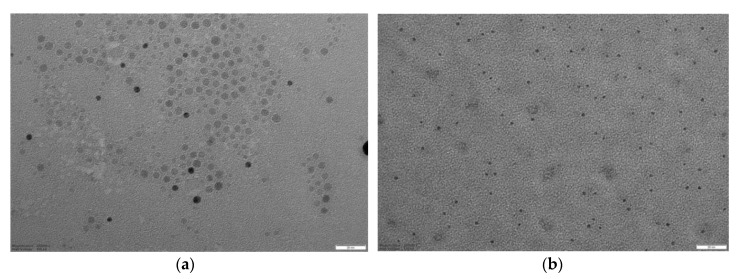

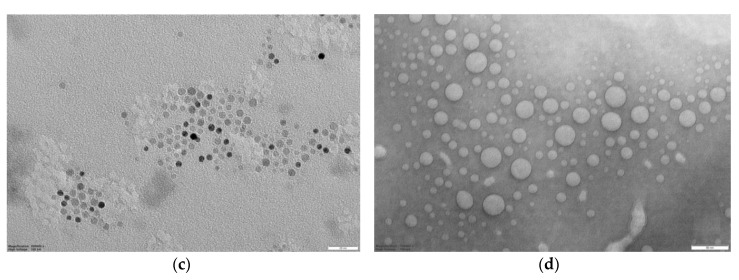
TEM images; (**a**) MIP 4VP-TY (200,000×, bar 20 nm); (**b**) MIP 4VP-HT (200,000×, bar 20 nm); (**c**) MIP 13-TY (200,000×, bar 20 nm); (**d**) MIP 13-HT (100,000×, bar 50 nm).

TEM analysis was performed on the best performing MIPs, namely 4VP-TY, 4VP-HT, 13-TY and 13-HT (Figure 3). More TEM images are reported in Supplementary Materials (Figures S13–S15). A population of by far smaller nanoparticles can be actually observed, together with a minor fraction of aggregates. The nanoparticles observed by TEM are actually smaller than 5 nm, with very low polydispersity, with the exception of MIP 13-HT which shows an average size of 13 nm and a clearly larger polydispersity.

### 3.3. Rebinding and Selectivity–Non-Fluorescent MIPs

**Figure 4:**
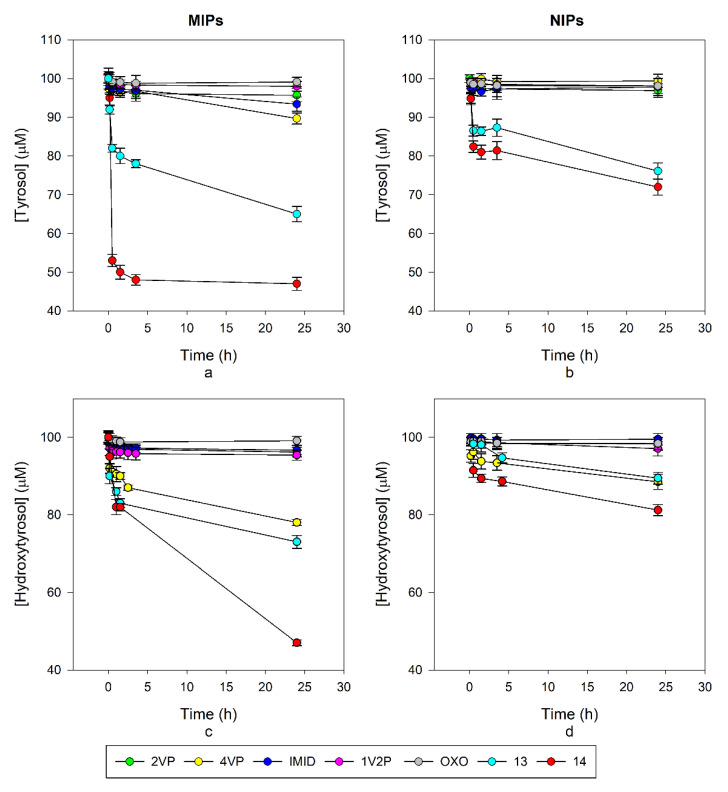
(**a**) Decay of TY concentration in 100 μM solutions upon incubation with the MIPs; (**b**) decay of TY concentration in 100 μM solutions upon incubation with the NIPs; (**c**) decay of HT concentration in 100 μM solutions upon incubation with the MIPs; (**d**) Decay of HT concentration in 100 μM solutions upon incubation with the NIPs.

Rebinding tests were carried out by HPLC (Table 2), first on the non-fluorescent MIPs; the best performing polymer for TY was 4VP-TY with about 10 nmol/mg ligand captured and an imprinting factor (IF, measured in comparison to the corresponding non-imprinted polymers, NIPs) of 17.2 after 24 h, while for HT, MIP 4VP-HT gave 22 nmol/mg rebinding although with an IF of 1.9 after 24 h. 4VP was identified as the best performing monomer both considering the rebinding capability and the specificity measured by the IF. Actually, these results reflect the interactions observed by ^1^H-NMR titrations where the major variations in the chemical shift for tyrosol were observed with 4VP, while for hydroxytyrosol the best results were obtained with 2VP as the monomer. Differences in the rebinding properties have been observed during time, such as in the case of 4VP-TY which triples up the rebinding capacity between 210 min and 24 h. Additionally, 4VP-HT and IMID-HT doubled up the value in the same time as can be observed from Table 2. In Figure 4a,c, the decay of phenols concentration in samples treated with the imprinted polymers are reported, while Figure 4b,d show the decay of phenols concentration in samples treated with the corresponding non imprinted polymers (NIP). The residual concentrations of phenols were calculated from the HPLC results. The amount of rebound phenols listed in Table 2 were calculated by the variation from the initial amount in the test solutions. Despite the fact that in these three cases the rebinding properties enhanced over time, the IF changed differently. With 4VP-TY and 4VP-HT the IF also increased over time, indicating the formation of specific sites in the polymer which contributed significantly to the rebinding properties in a relatively slow process. In the case of IMID-HT, the IF value decreased over time, indicating that non-specific interactions occur in prevalence and an equal rebinding capacity is observed for MIP and NIP (IF = 1). OXO MIPs showed an IF < 1, with a rebinding capacity of the corresponding NIP major than the one for the MIP itself. This first series of evaluation led us to select the 4VP monomer for further developments.

Besides rebinding and specificity, 4VP-TY and 4VP-HT were evaluated also for selectivity. 4VP-TY was evaluated in rebinding of HT, and proved to be selective for its template as rebinding of TY was about 4-times higher than that of HT (4.0 nmol/mg vs. 1.0 nmol/mg). Conversely, 4VP-HT was not selective when the experiment was carried out at 50 μM ligands (2.3 nmol/mg for both), but became selective towards HT when the measure was repeated at 100 μM phenols (3.2 nmol/mg vs. 2.3 nmol/mg). The higher selectivity of the 4VP-TY MIP could be probably explained by the smaller size of TY compared to HT, which bears an additional hydroxyl group at the aromatic ring. The selectivity of 4VP-HT towards its template is higher (~8 times) than the one of 4VP-TY towards its template (~5 times) (Table 2). This is probably due to the formation of a hydrogen bonds network around HT.

### 3.4. Fluorescent MIPs

**Figure 5:**
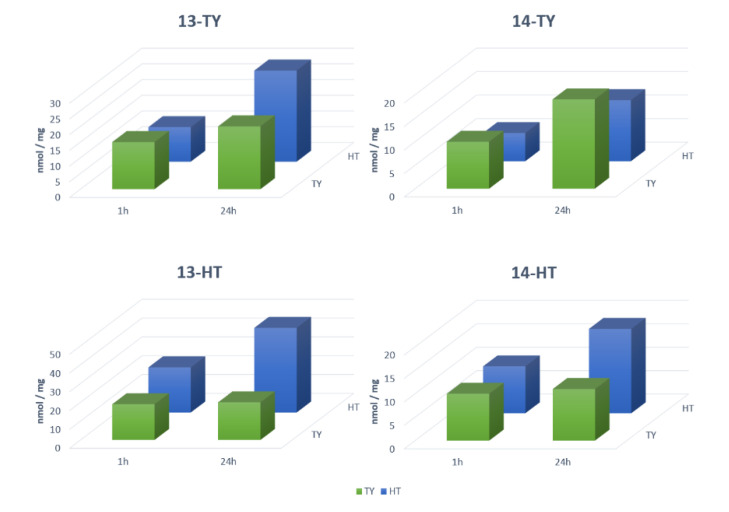
Competition tests for MIPs 13-TY, 14-TY, 13-HT and 14-HT towards the templates TY and HT.

This first series of results, although promising, was not completely satisfactory as the absolute rebinding activity of the materials is good, but not excellent in comparison with literature data on similar polymers [18]. We have therefore decided to keep 4VP as the functional monomer while moving to fluorescent MIPs, but to change the cross-linker, choosing DVB for the last four imprinted polymers, in the hope to improve rebinding by the introduction of its further aromatic system. In the fluorescent materials, the fluorescent co-monomers **13** and **14** replaced NIPAM in the same % composition as reported in Table 1. The resulting rebinding capacity was fully satisfactory: MIPs 13-TY and 13-HT showed a rebinding of 30–35 nmol/mg, and in MIPs 14-TY and 14-HT the value was over 50 nmol/mg in both cases, although the specificity was less favorable with IF not higher than 2.7 (Table 2). Nevertheless, this value is still a good indication of the occurrence of specific sites inside the imprinted material, and sufficient to further developing sensing polymers. To this point, the fluorescent materials were designed to be used as sensors in analytical protocols to be exploited in real samples containing HT, TY and OL together, and we have therefore evaluated their selectivity in competition experiments by testing the binding capacity in solutions containing both TY and HT either 50 or 100 μM (Figure 5).

MIP 13-TY shows a significant selectivity at 100 μM ligands towards its template during the first 60 min of incubation, after this time, selectivity decreased, and at 24 h it is actually reverted in favor of HT. MIP 13-HT is instead selective for HT even at 24 h, and its selectivity increases with the incubation time (Table 2). Additionally, MIPs 14-TY and 14-HT are selective towards their ligands, and the selectivity is constant all along the rebinding kinetics (Table 2). The selectivity is not significantly affected by the concentrations of the two ligands. In conclusion, the addition of fluorescein as the monomer and the replacement of MBA as the crosslinker with DVB enhances the possibility to have π-π stacking leading to a higher rebinding towards both templates although with a lower specificity and selectivity. Since our aim was the recognition of OL, which is principally present in olive leaves extract, a lower selectivity could represent an advantage. The rebinding of OL was also evaluated on MIPs 13-TY and 13-HT, since, as it will be shown in the next paragraph, they are the best performers in fluorimetry. OL is the most abundant phenolic compound in olive leaves extracts and its concentration is often 1–2 orders of magnitude higher than TY and HT. For this reason, its rebinding was studied at higher concentration, namely 250 μM. Both MIP 13-TY and 13-HT performed in an excellent way, being able to remove almost completely OL from the test solution in less than one-hour incubation. This result is very important, as it confirms our starting hypothesis on the possibility of using HT and TY as templates to develop MIPs capable to rebind OL.

### 3.5. Fluorimetry

**Figure 6:**
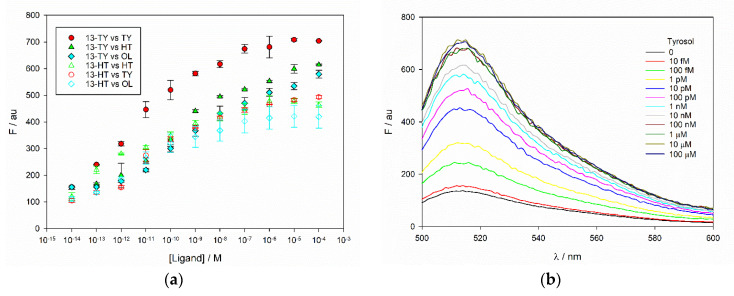
(**a**) fluorescence emission of MIPs 13-TY and 13-HT upon increasing concentrations of TY, HT and OL. (**b**) emission spectra of MIP 13-TY along the titration with TY.

The promising properties measured in the rebinding tests were confirmed by fluorimetry. The pH of the aqueous extracts of olive leaves as obtained by our protocol is 5.6, and we have measured an average conductivity of 3.6 mS/cm, similar to that usually found in plant aqueous extracts and in physiological buffers. The pH of a colloidal solution of our fluorescent MIPs at 2.5 μg/mL in water is 6.6. To take into account potential effects of pH and ionic strength, the polymers were tested under different conditions, namely in water as 2.5 μg/mL colloidal solutions, in 100 mM citrate buffer at pH 5 (below that of real samples and at similar ionic strength), and in PBS buffer at pH 7.5 (a value over the pH of real samples and over the third pK_a_ of fluorescein). TY, HT and OL were added to the MIP in a range of concentrations spanning from 10 fM to 100 μM. The amount of MIP was nearly 3-orders of magnitude less than that used in the rebinding assay, as the fluorescence emission was strong enough to allow working at very low concentrations. Under these conditions, a 10 min incubation time was sufficient to obtain stable changes in emission. Fluorescence was monitored at 480 nm excitation and 515 nm emission and, as expected, it did increase in citrate buffer, and in water. MIPs 13-TY and 13-HT resulted much more sensitive, and their response curves in water are reported in Figure 6. The emission spectrum of MIP 13-TY upon titration with TY is reported in Figure 6 as an example, showing the increase of the emission peak, without changes of its shape and drifts of the emission maximum, thus supporting the evidence that the phenomenon is related to a true activation of the emission. The same was observed also in all the other titrations. The overall change is nevertheless larger in water at pH 6.6 than in citrate buffer at pH 5. Conversely, only a negligible change was observed when the titrations were carried out at pH 7.5 in PBS buffer, where the initial emission of the MIPs is much higher (Supplementary Materials, Figures S16 and S17).

The whole change occurs at very low concentration of phenols, in a range between 100 fM and 100 nM. This ensures that it is not influenced by changes in pH, that are actually not detectable also in water after the addition of 100 nM targets. Before showing in the last section the potential of such system for the detection of our phenols, we discuss the possible origin of the activation of fluorescein emission. At pH 7.5, the dianion of fluorescein predominates in solution, representing, on the basis of the pK_a_, about the 83% of the total fluorophore. The dianion is more fluorescent than the monoanion, which is the most abundant species at both pH 6.6 (84%) and 5 (77%). As no changes in emission are observed at pH 7.5, we can exclude that the activation is due to an interaction of the phenols with the dianion. The monoanion seems to be rather involved, and the interaction with the phenols inside the MIP could actually shift the lactone-quinone equilibrium of the monoanion towards the fluorescent quinone form, as envisaged in our starting hypothesis, or, more unlikely, could shift the equilibrium monoanion/dianion towards the dianion.

Both the polymers are sensitive to the whole set of tested ligands. In this evaluation, MIP 13-TY is more selective towards its template TY, while HT and OL give a very similar response. Although the absolute increase of signal is higher in 13-TY, also 13-HT performs well and the sensitivity is very high. It is less selective than 13-TY, as it could be expected from the structure of its ligand, and the interactions with the three phenols leads to similar results.

The titration curves of Figure 6 could be in principle exploited as calibration curves for the detection of phenols. To this end, several analytical parameters have been evaluated and reported in Table 3.

The limits of detection are always below 1 pM for all the phenols, and the limits of quantification, defined as the minimum concentration giving a signal over the one measured in the absence of analyte plus 10 times its standard deviation, are in the 0.2–10 pM range. Such values are satisfactory as to the possibility of evaluating the concentrations of ligands in real olive leaf extracts, where the expected concentrations are much higher, especially in the case of OL. The titrations are well reproducible, with precisions on the read emissions within 5%, with the exception of OL titration with 13-HT.

### 3.6. Performance on Real Samples

**Figure 7:**
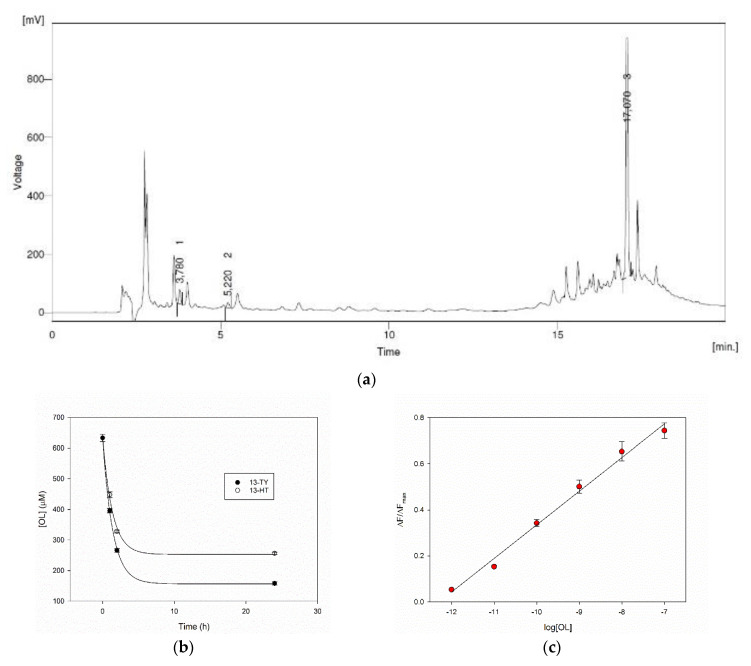
(**a**) HPLC analysis of olive leaves extracts, (**b**) capture of OL from olive leaf extracts after addition of MIPs, (**c**) calibration curve of fluorescence enhancement after addition of OL to MIP 13-TY (r^2^ = 0.991, intercept 1.796 ± 0.067, slope 0.1461 ± 0.0069).

The HPLC record of a real sample of olive leaves aqueous extract is reported in Figure 7a. The natural matrix delivers a complex chromatogram, where the peaks corresponding to TY, HT and OL were identified at 5.2 min, 3.8 min and 17.1 min respectively, thanks to the addition of standards. Their concentrations have been evaluated by HPLC using calibration curves, and in this typical sample, a 2.0, 4.5 and 633 μM concentrations of TY, HT and OL were found respectively.

The rebinding ability of our MIPs was therefore evaluated on the extract. The amount of MIPs 13-TY and 13-HT to be added to the samples was defined according to its ability to capture OL (Table 2), as this is the main compound in the extract. We have found that the polymers at 6 mg/mL, in a 1:1 dilution of the extract, are able to remove more than 70% of OL with a very rapid process during the first two hours (Figure 7b). The possibility of measuring the concentration of phenols on the real samples was finally evaluated with MIP 13-TY as its calibration curve performs at best, on a sample containing 398 μM OL. This concentration is well over the upper limit of the dynamic range of our system. This fact allows us to highly dilute the sample minimizing the matrix effect. The sample was, in fact, added at a final 1:10^5^ dilution to a 2.5 mg/mL solution of MIP 13-TY. Under this dilution the pH is 6.6 as in the water solution of MIP, and the system behaves as shown before in water. The increase of fluorescence was recorded. The datum from a triplicate measure was used to evaluate the total concentration of phenols expressed as OL, and to this end, a calibration curve was used from a linear regression of the fluorescence titration data in the 1 pM–100 nM range (Figure 7c). By this way, the calculated phenol concentration in the real sample turned out to be 433 ± 20 μM. This result is in line with that obtained in the reference HPLC method.

## 4. Conclusions

In conclusion, we have shown that molecularly imprinted nanoparticles embedding fluorescein and carefully selected functional monomers and cross-linkers can be used to obtain a sensing material where the signaling mechanism is based on the recognition of the target phenol. The high sensitivity allows their use in real samples, diluted enough to ensure minimization of matrix effects and potential competition from other compounds, on our proof of concept measure. The exploitation of such materials in portable sensing devices can be planned as a development of this work, after a full evaluation of a wide set of real samples, and we are currently working towards this goal.

## Figures and Tables

**Table 1:** Composition of the polymerization mixtures for monomers **5**–**9** and **13**–**14**.

Polymer	Template [mg]	Functional Monomer [mg]	AIBN [mg]	Comonomer [mg]	Crosslinker [mg]	DMSO [mL]
2VP-TY	TY 8.3	2VP 5.3	16.5	NIPAM 5.7	MBA 36.0	4.23
2VP-HT	HT 9.2	2VP 5.3	16.5	NIPAM 5.7	MBA 36.0	4.23
4VP-TY	TY 8.3	4VP 5.3	16.5	NIPAM 5.7	MBA 36.0	4.23
4VP-HT	HT 9.2	4VP 5.3	16.5	NIPAM 5.7	MBA 36.0	4.23
1V2P-TY	TY 8.3	1V2P 5.6	16.5	NIPAM 5.7	MBA 36.0	4.26
1V2P-HT	HT 9.2	1V2P 5.6	16.5	NIPAM 5.7	MBA 36.0	4.26
IMID-TY	TY 8.3	IMID 4.7	16.5	NIPAM 5.7	MBA 36.0	4.18
IMID-HT	HT 9.2	IMID 4.7	16.5	NIPAM 5.7	MBA 36.0	4.18
OXO-TY	TY 8.3	OXO 5.7	16.5	NIPAM 5.7	MBA 36.0	4.27
OXO-HT	HT 9.2	OXO 5.7	16.5	NIPAM 5.7	MBA 36.0	4.27
13-TY	TY 8.7	4VP 5.6	20.2	**13** 23.6	DVB 30.0	5.89
13-HT	HT 8.3	4VP 5.5	20.7	**13** 23.5	DVB 30.0	5.89
14-TY	TY 8.4	4VP 7.0	20.8	**14** 16.7	DVB 30.0	5.14
14-HT	HT 8.5	4VP 5.4	20.6	**14** 16.9	DVB 30.0	5.14

**Table 2:** Characterization of the imprinted materials.

MIP	F.M. 15% mol	C.M. 15% mol	C.L. 70% mol	Yield MIP % ^a^	Yield NIP % ^a^	Size ^b^ nm	PDI	Rebinding ^c^ nmol/mg			IF ^c^
								TY	HT	OL	
2VP-TY	5	10	11	75	91			4.3 ± 0.2 (3.9 ± 0.2)			1.4 (1.4)
2VP-HT	5	10	11	73					3.8 ± 0.2		2.2
4VP-TY	6	10	11	90	85	4.85 ± 0.65	0.135	10.3 ± 0.3 (3.3 ± 0.1)	2.2 ± 0.3		17.2 (4.1)
4VP-HT	6	10	11	90		2.40 ± 0.33	0.136	2.6 ± 0.1	22.0 ± 1.3 (13.1 ± 1.5)		1.9 (1.1)
1V2P-TY	7	10	11	87	84			2.0 ± 0.3			1.1
1V2P-HT	7	10	11	95					3.3 ± 0.2		1.1
IMID-TY	8	10	11	96	89			4.6 ± 0.5 (4.2 ± 0.4)			2.1 (1.5)
IMID-HT	8	10	11	86					6.6 ± 0.5 (3.0 ± 0.1)		1 (4.3)
OXO-TY	9	10	11	61	74			0.6 ± 0.07			-
OXO-HT	9	10	11	50					0.7 ± 0.05		-
13-TY	6	13	12	74	72	4.20 ± 0.65	0.154	35 ± 2		>250 ^§^	1.5
13-HT	6	13	12	80		12.97 ± 5.60	0.432		28 ± 3	>250 ^§^	1.4
14-TY	6	14	12	70	74			52 ± 3			1.9
14-HT	6	14	12	70					51 ± 3		2.7

F.M., functional monomer; C.M., co-monomer; C.L., cross-linker; PDI, polydispersity index (ratio between the standard deviation of the particle size and the average particle size); ^a^ in weight over the total mass of monomers; ^b^ from analysis of TEM images; ^c^ from HPLC measure at 24 h on 100 μM solutions containing 1 mg/mL molecularly imprinted polymer (MIP). In brackets: at 210 min; ^§^ from 250 μM solutions of OL.

**Table 3:** Sensitivity and precision of MIP fluorescence titrations.

	13-TY			13-HT		
	LOD ^a^, pM	LOQ ^b^, pM	Precision ^c^, %	LOD ^a^, pM	LOQ ^b^, pM	Precision ^c^, %
TY	0.1	0.2	2.64	0.5	5.4	4.05
HT	0.5	1.2	4.35	0.5	1.3	3.06
OL	0.9	2.1	3.12	0.7	10.2	8.92

^a^: limit of detection evaluated with the 3σ method after linear regression of emission data vs. log of ligand concentrations in the 10 fM–10 nM range; ^b^: limit of quantification set at 10σ over the starting signal with no ligands; ^c^: average % error over the whole set of standard deviations of the emission signals from triplicate measures at each concentration.

## Supplementary Materials

The following are available online at https://www.mdpi.com/2079-4991/10/6/1011/s1, HPLC calibration curves, NMR titrations, TEM images and fluorescence spectra.

## Abbreviations

AIBN	2,2′-azobisisobutyronitrile
DVB	1,4-Divinylbenzene
DFT	Density Functional Theory
EFSA	European Food Safety Authority
HT	Hydroxytyrosol
IMID	1-vinylimidazole
LDL	Low Density Lipoprotein
MBA	*N,N**′*-methylenebisacrylamide
NIPAM	*N*-isopropylacrylamide
OL	Oleuropein
OXO	4-vinyl-1,3-dioxolan-2-one
pcm	Polarizable continuum model
SERS	Surface Enhanced Raman Scattering
SPE	Solid Phase Extraction
TY	Tyrosol
1V2P	1-vinyl-2-pyrrolidinone
2VP	2-Vinylpyridine
4VP	4-vinylpyridine
