# Supplementary material for: Signal-On Fluorescent Imprinted Nanoparticles for Sensing of Phenols in Aqueous Olive Leaves Extracts
Source: Nanomaterials (Basel). 2020 May 26;10(6):1011. doi: 10.3390/nano10061011 (PMC7353427; doi:10.3390/nano10061011)
Supplement: Supplementary file 1 [file nanomaterials-10-01011-s001.pdf]

Supplementary Materials

Signal-On Fluorescent Imprinted Nanoparticles for Sensing of Phenols in Aqueous Olive Leaves Extracts

Ada Stavro Santarosa, Federico Berti, Martina Tommasini, Antonella Calabretti and Cristina Forzato *

Dipartimento di Scienze Chimiche e Farmaceutiche, Università degli Studi di Trieste, via Giorgieri 1, 34127 Trieste, Italy; adastavro@gmail.com (A.S.S.); fberti@units.it (F.B.); martina.tommasini28@gmail.com (M.T.); ANTONELLA.CALABRETTI@deams.units.it (A.C.)

* Correspondence: cforzato@units.it; Tel.: +39-040-558-3921

Calibration Curves

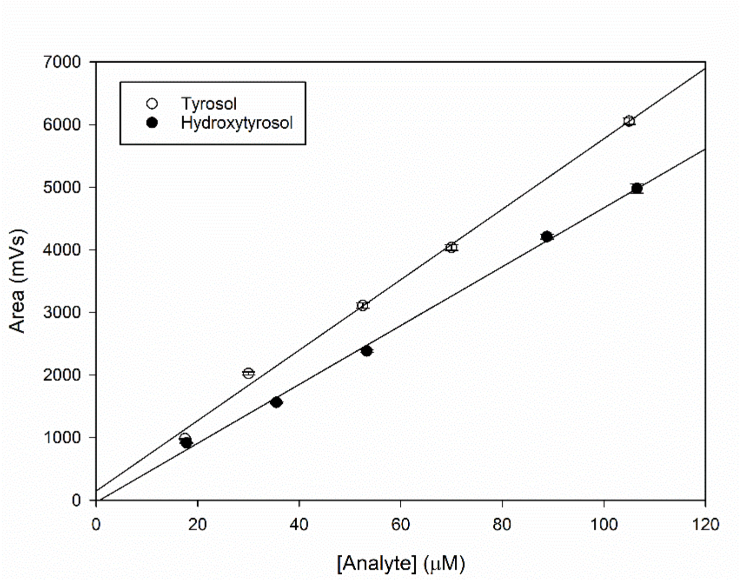

Figure S1. HPLC calibration for TY and HT, average of triplicate measures. Linear regression for TY: r^2 0.992, slope 56.29, intc. 146.37. Linear regression for HT: r^2 0.997, slope 47.01, intc. −29.15.

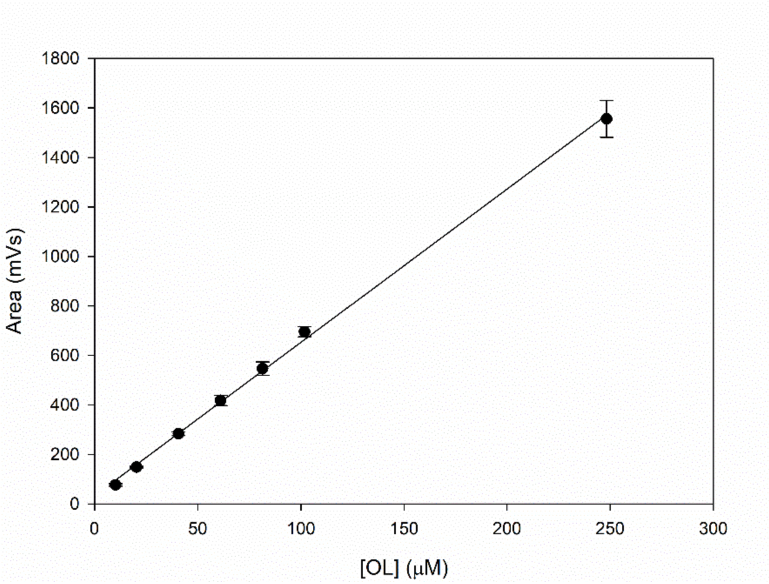

Figure S2. HPLC calibration for OL, average of triplicate measures. Linear regression: r^2 0.9988, slope 6.198, intc. 32.80.

Interactions of Functional Monomers with the Targets: NMR Titrations

Figures S3–S8 reported the most significant changes in the NMR spectra of TY and HT upon titration with the functional monomers leading to the most relevant effect (2-VP, 4-VP and IMID).

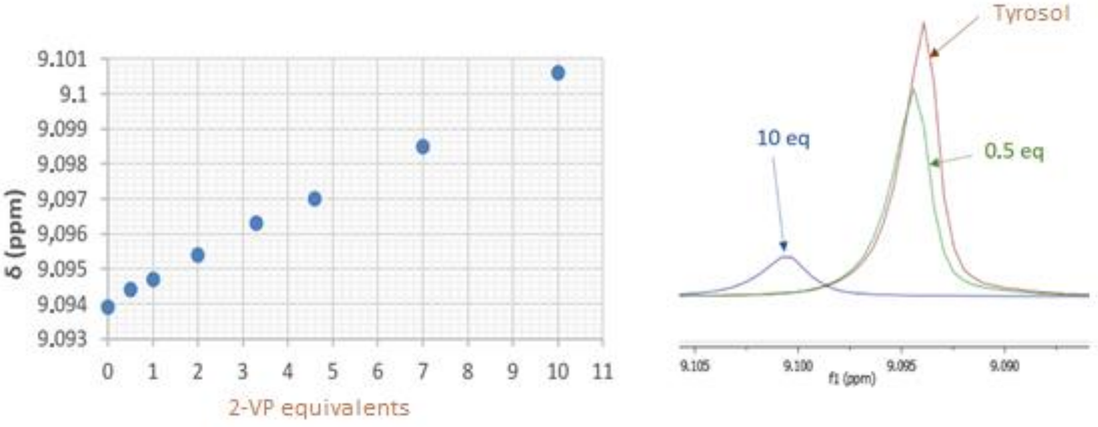

Figure S3. Shift of the phenolic proton signal of TY upon titration with 2-VP.

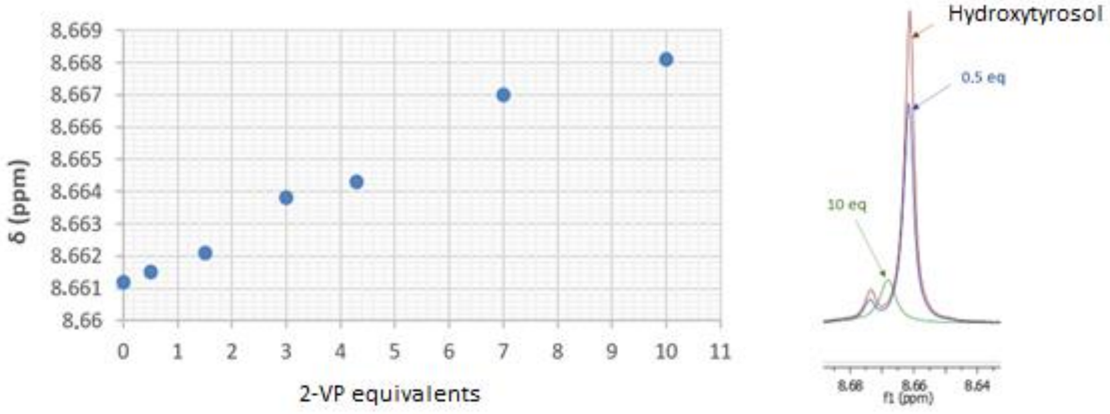

Figure S4. Shift of the C3-phenolic proton signal of HT upon titration with 2-VP.

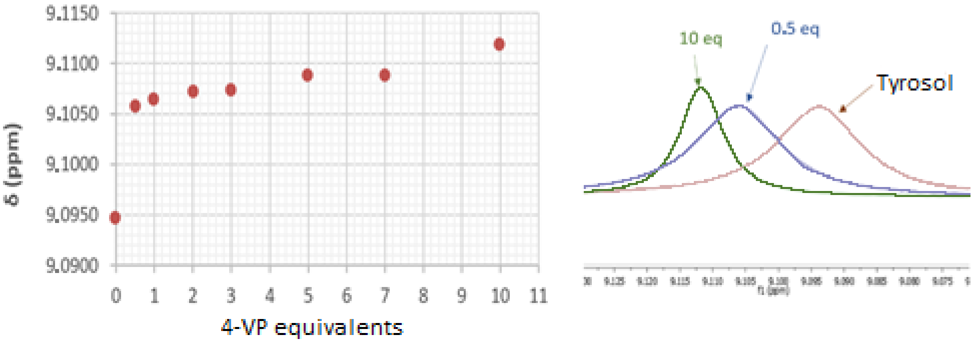

Figure S5. Shift of the phenolic proton signal of TY upon titration with 4-VP.

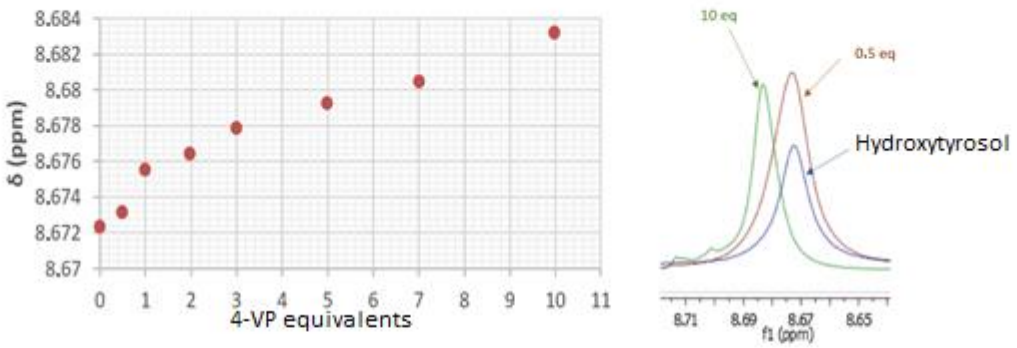

Figure S6. Shift of the C3-phenolic proton signal of HT upon titration with 4-VP.

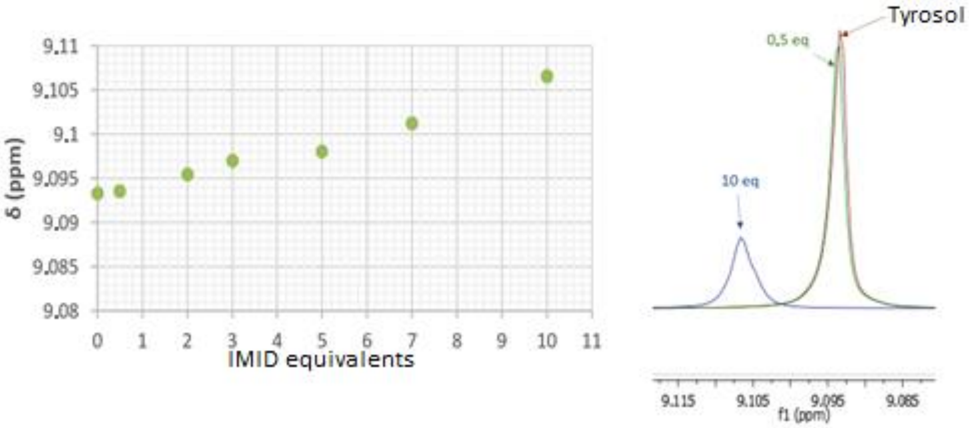

Figure S7. Shift of the phenolic proton signal of TY upon titration with IMID.

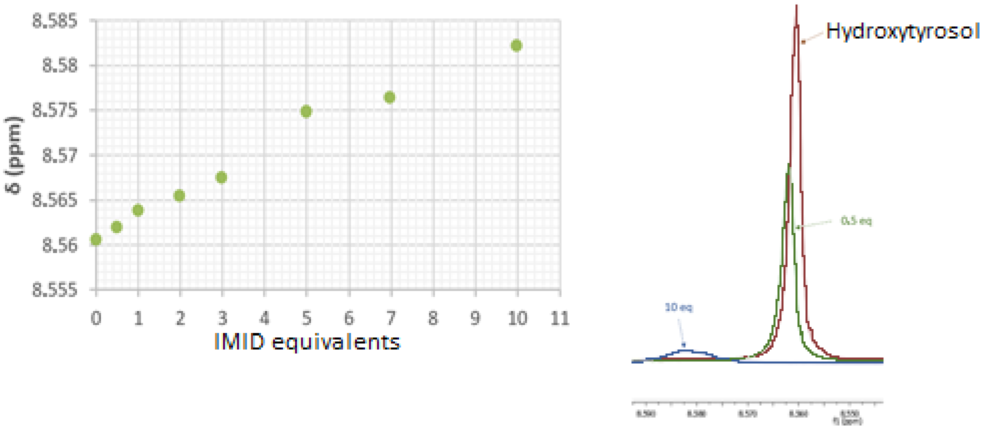

Figure S8. Shift of the C3-phenolic proton signal of HT upon titration with IMID.

Figures S9–S12 reported the change in chemical shift of TY, HT and OL proton NMR signals upon addition of 10 equivalents of functional monomers.

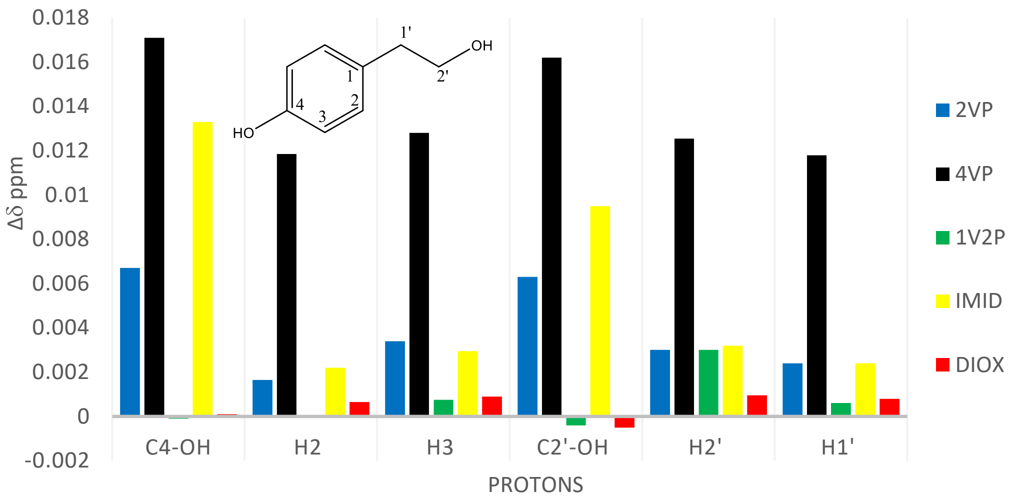

Figure S9. ^{1}H-NMR titrations of **1** with the monomers **5–9.**

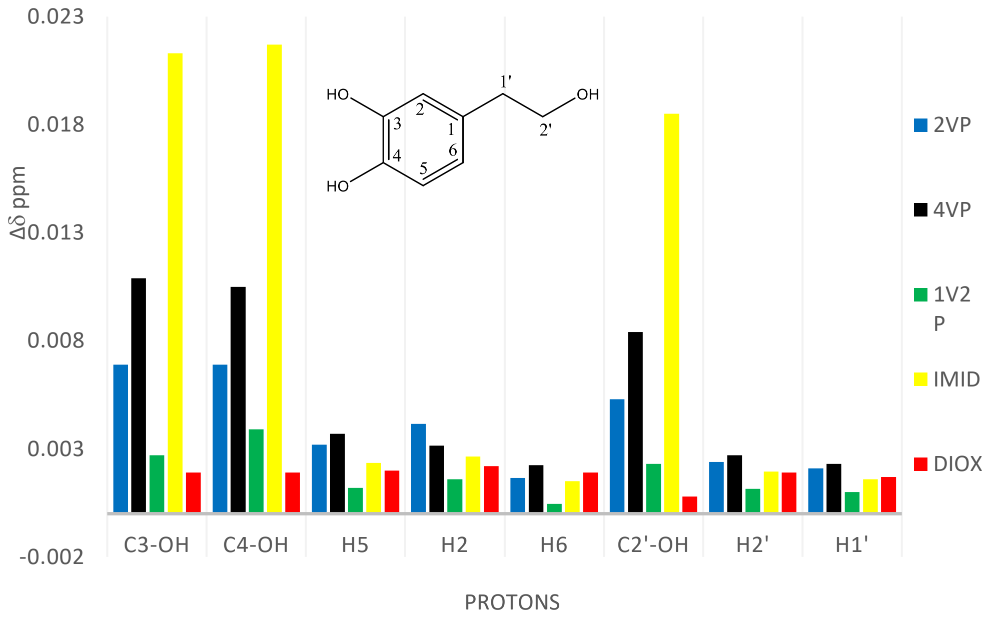

Figure S10. ^{1}H-NMR titrations of **2** with the monomers **5–9.**

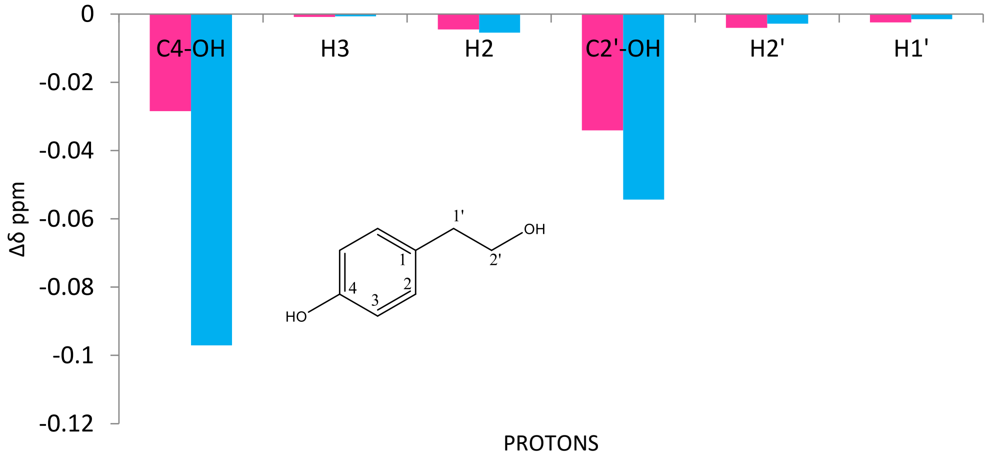

Figure S11. ^{1}H-NMR titrations of **1** with the comonomers **13** (blue) and **14** (magenta).

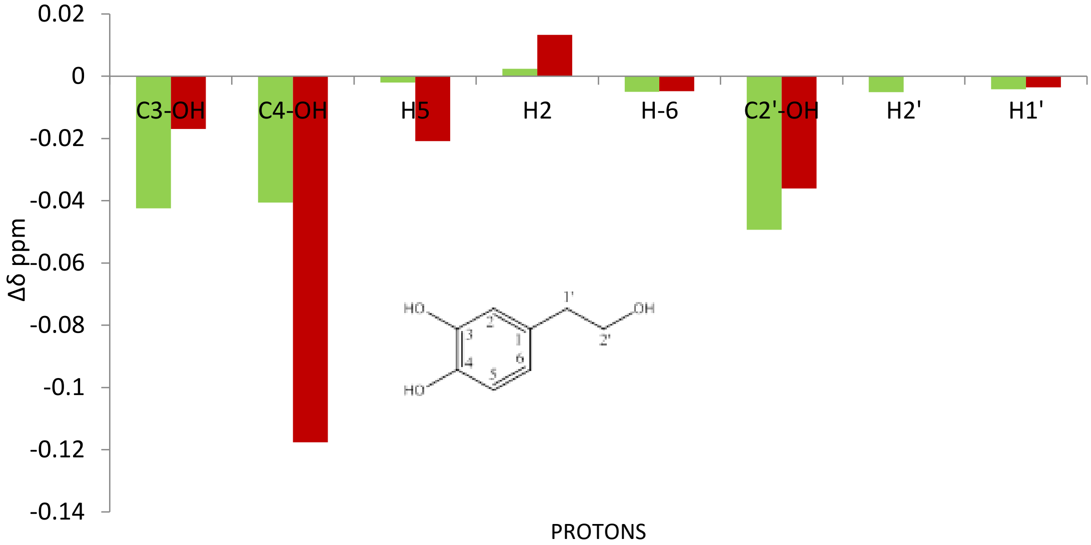

Figure S12. ^{1}H-NMR titrations of **2** with the comonomers **13** (red) and **14** (green).

TEM Images

TEM images reported in Figures S13–S15 show the occurrence of aggregate fractions of the MIPs, besides the nanoparticle populations shown in the main text.

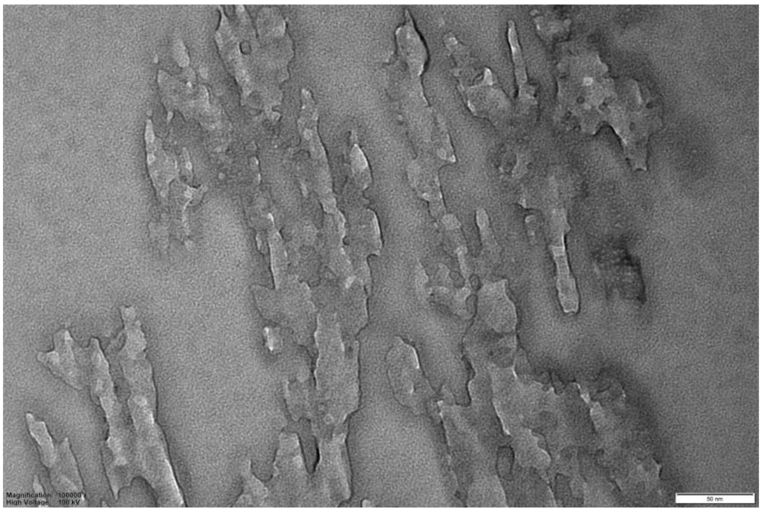

Figure S13. Laminar aggregates of 4VP-HT. Bar: 50 nm.

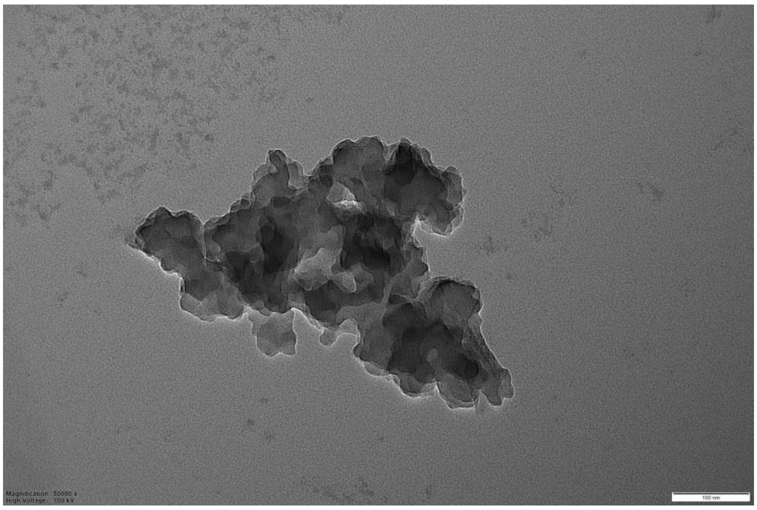

Figure S14. Aggregate of 13-TY. Bar: 100 nm.

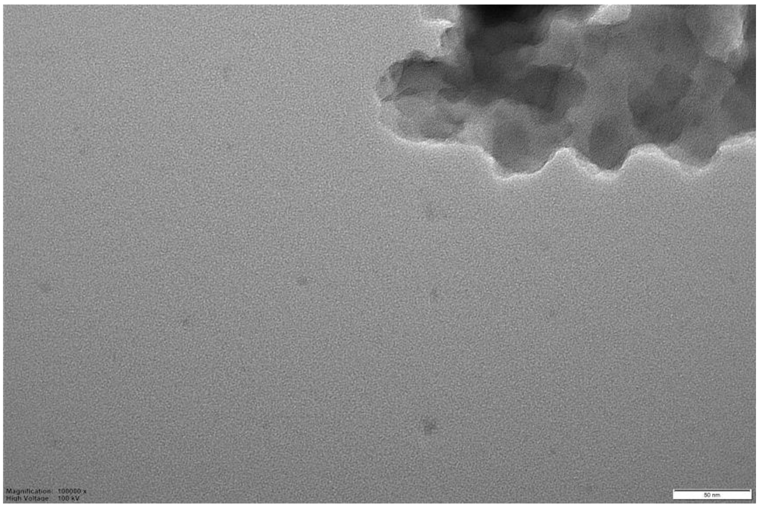

Figure S15. Aggregate of 13-HT. Bar: 50 nm.

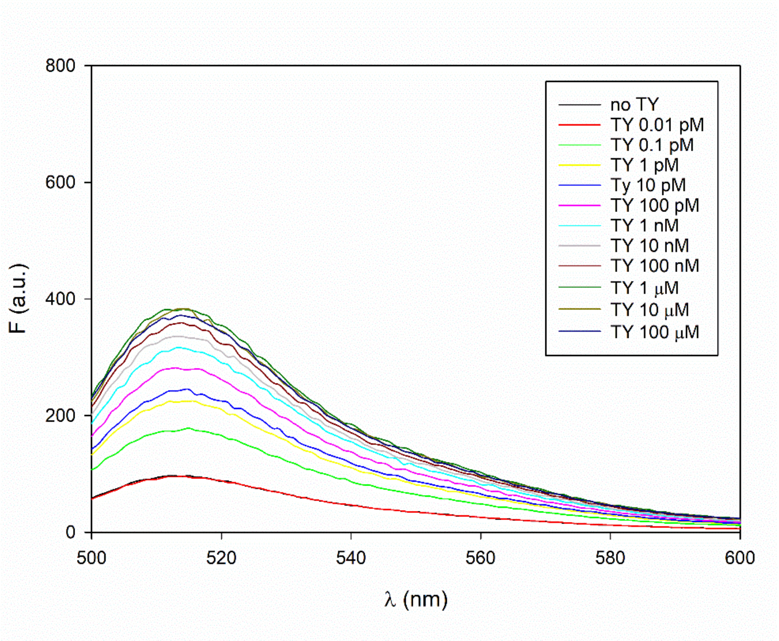

Figure S16. Emission spectra of MIP 13-TY along the titration with TY in citrate buffer pH 5.0.

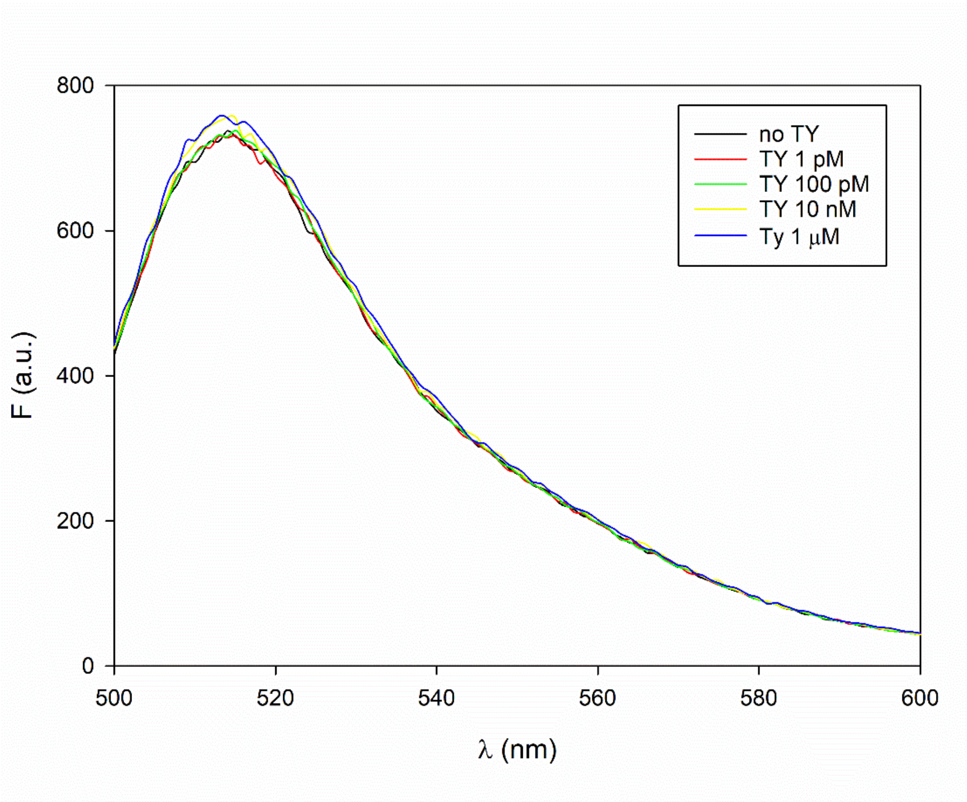

Figure S17. Emission spectra of MIP 13-TY along the titration with TY in PBS buffer pH 7.5.
